# Creating a sustainable and inexpensive dry animal training model for liver surgery in low- and middle-income countries

**DOI:** 10.3332/ecancer.2025.1911

**Published:** 2025-05-27

**Authors:** Jeanine Justiniano, Ally H Mwanga, Daniel W Kitua, Nashivai E Kivuyo, Seif Wibonela, Cameron E Gaskill

**Affiliations:** 1 Department of Surgery, University of California Medical Center, Sacramento, CA, USA; 2 Department of Surgery, Muhimbili University of Health and Allied Sciences, Dar es Salaam, Tanzania; 3 Surgical Skills Laboratory, Muhimbili University of Health and Allied Sciences, Dar es Salaam, Tanzania

**Keywords:** animals, hepatectomy, simulation training, Sub-Saharan Africa, cost-benefit analysis

## Abstract

**Background:**

The growing global demand for surgical simulation training is particularly challenging in low- and middle-income countries (LMICs), where access to advanced technology is limited. Despite increasing demand for these services, hepatobiliary surgical training in LMICs is constrained by a need for more training facilities and experts. We, therefore, designed a feasible, cost-effective liver surgery training model using bovine liver. We hypothesise that this sustainable model can significantly enhance surgical training in LMICs.

**Methods:**

A bovine liver was procured from a local slaughterhouse, with careful preservation of its vascular structures. The specimen was transported to the Muhimbili University of Health and Allied Sciences’ Laboratory and prepared using a back-table technique. Major vessels were connected to a water flow system, simulating near-physiological central venous and arterial pressures. These parameters were adjusted to mimic real-time effects, creating a training environment similar to that of an actual surgical candidate.

**Findings:**

A partial hepatectomy was successfully performed using the crush-clamping technique. Hydrodynamic alterations and simulated bleeding were effectively managed through the Pringle maneuver, suture ligation and stick-tying techniques. The procedure was completed in approximately 1 hour, with an estimated blood loss of 700 mL.

**Conclusion:**

An inexpensive, ethical and sustainable bovine liver model was designed for surgical training. This simulation can be easily replicated in training facilities across LMICs to enhance surgical education, particularly in hepatobiliary surgery.

## Background

Liver cancer, or hepatocellular carcinoma (HCC), is the third leading cause of cancer-related mortality worldwide, with over 866,000 cases, nearly 80% of which occur in low- and middle-income countries (LMICs) [1]. In sub-Saharan Africa (SSA), 46,000 new liver cancers are occurring each year, estimated to increase by 2-fold by 2040. While curative treatments for HCC may include liver transplantation and/or ablation, surgical resection remains the primary modality within the capacity of low-resource settings. However, despite the increasing demand for surgical hepatobiliary services, there remains a disparity between the number of trained surgeons and the number of procedures performed.

Liver surgery is complex and technically demanding. Even in high-resource settings, perioperative mortality rates for hepatectomy can be as high as 20% [2]. The experience and training of the operating surgeon correlate directly with postoperative outcomes [3]. Adequate case volume and experience with liver surgery often require fellowship-level training, generally unavailable to surgical trainees in low-resource settings. Surgical simulation training has recently been proposed as a potential method to augment traditional operating-room-based surgical training. The benefits of simulation include providing a safe environment where a trainee can acquire repetitive experience and technical errors can be made without clinical consequences.

Tanzania is working toward a comprehensive liver cancer treatment program [4]. As a component of this, a surgical training curriculum was developed to facilitate early faculty and surgical residents in gaining the knowledge and technical skills to perform liver resections. High-fidelity, locally available surgical models that mimic human anatomy and tissue character are essential for developing the fine motor skills and tactile sensitivity necessary for performing complex surgical procedures [5]. In the manuscript, we describe the development of a locally sourced, tissue-based bovine liver surgery model, whose anatomy closely mirrors human liver size and structure, for use in surgical skills simulation in SSA.

## Methodology

Working in collaboration with officials from a local stockyard, slaughterhouse employees were trained on bovine liver procurement to maintain intact anatomy. The liver was removed by dividing hepatic ligaments, incising supra- and infra-hepatic inferior vena cava (IVC) and dividing mesenteric vessels inferior to the pancreatic neck. The gallbladder and part of the duodenum were preserved, keeping the porta hepatis intact. The procured bovine liver was immediately perfused with tap water to remove the remaining blood in the intrahepatic vessels and prevent coagulation. The liver was kept refrigerated until the intended use. ‘Back-table’ dissection exposed inflow and outflow vessels.

**Figure 1.**
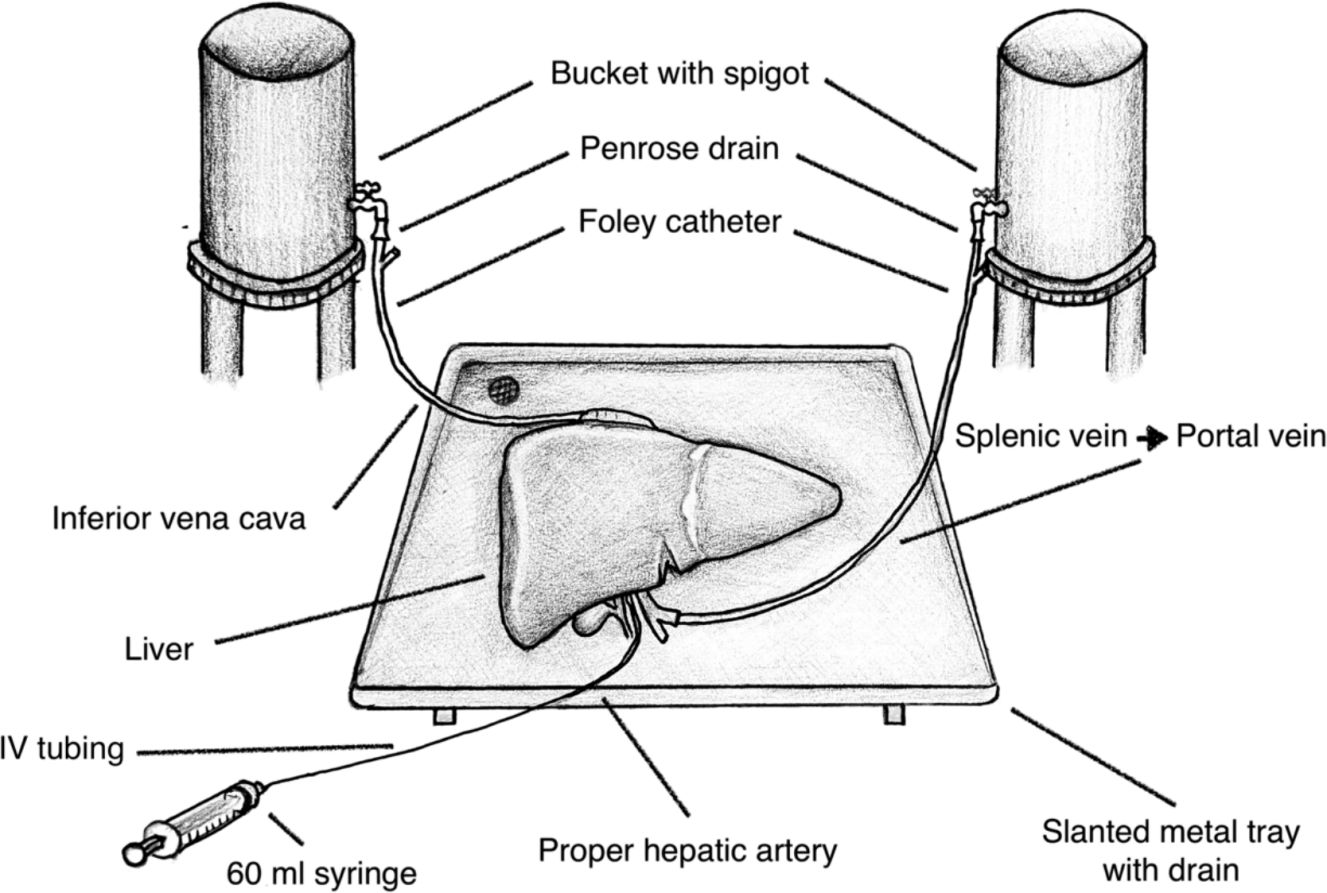
Illustration of the bovine liver training model.

**Figure 2.**
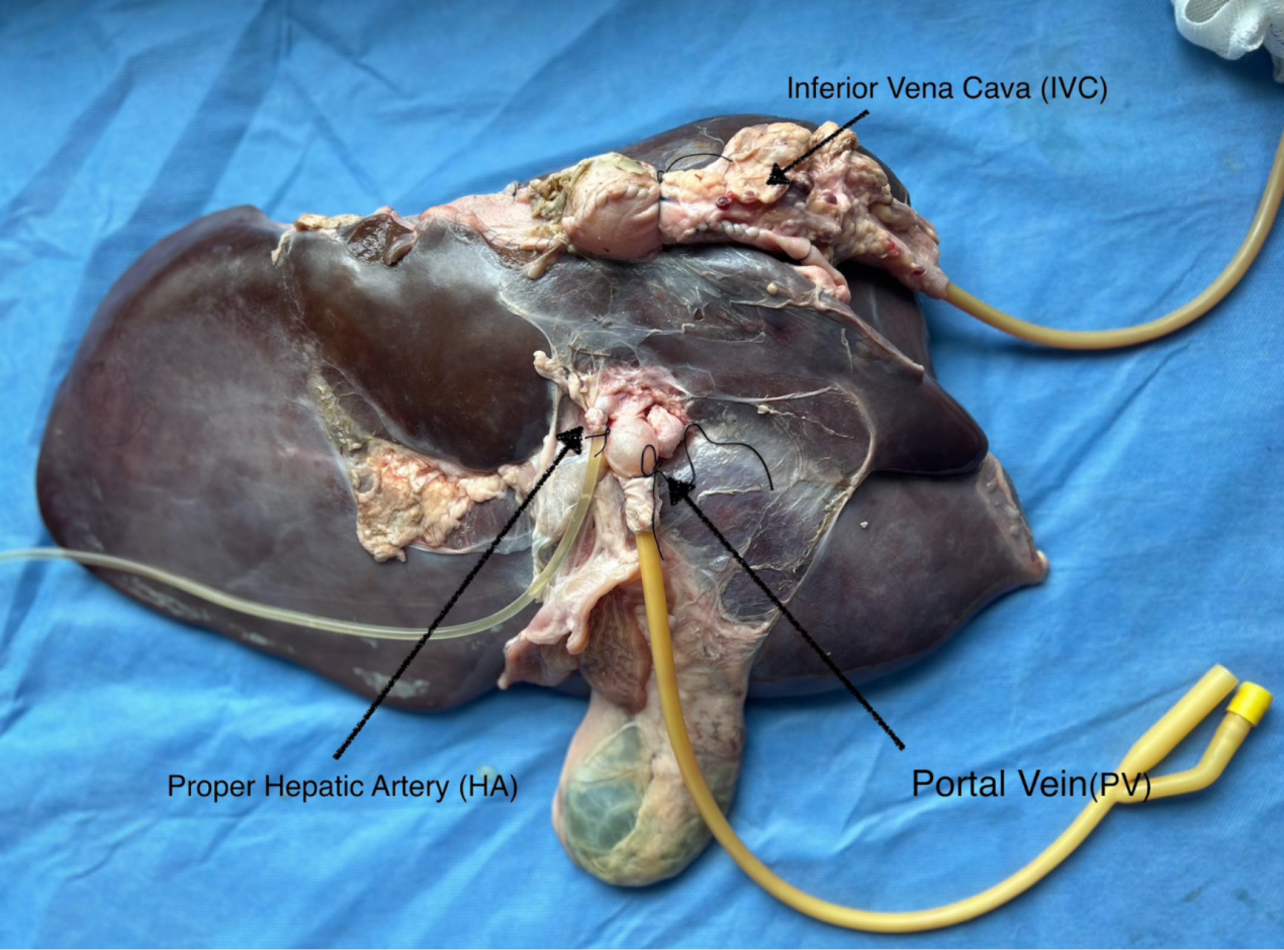
Catheterisation of the IVC, PHA and PV.

Using tools readily available from the university’s laboratory and the local supermarket listed in Table 1, we de‑vised a liver model with flowing colored water through the IVC, portal vein (PV) and proper hepatic artery (PHA) to simulate real-time bleeding (Figure 1). The splenoportal confluence was ligated, allowing catheterisation of the splenic vein with a Fogarty catheter. The PHA was catheterised with IV tubing. The infrahepatic IVC was ligated and the suprahepatic IVC was sewn around an additional Fogarty catheter (Figure 2). Each catheter was attached to fluid containers. PV and IVC catheter pressures were measured using an available transducer to calibrate the appropriate heights of the fluid containers to mimic portal and central venous pressures, respectively. Smaller branching vessels on the major inflow and outflow vessels were ligated to create a closed system. Clamps were applied to each catheter to allow increased control of ‘blood flow’ during trainee instruction. The laboratory manager monitored fluid container volume and simulated pressure throughout the exercise and would intermittently simulate arterial bleeding by injecting colored water into the PHA through a 60 mL syringe.

**Figure 3.**
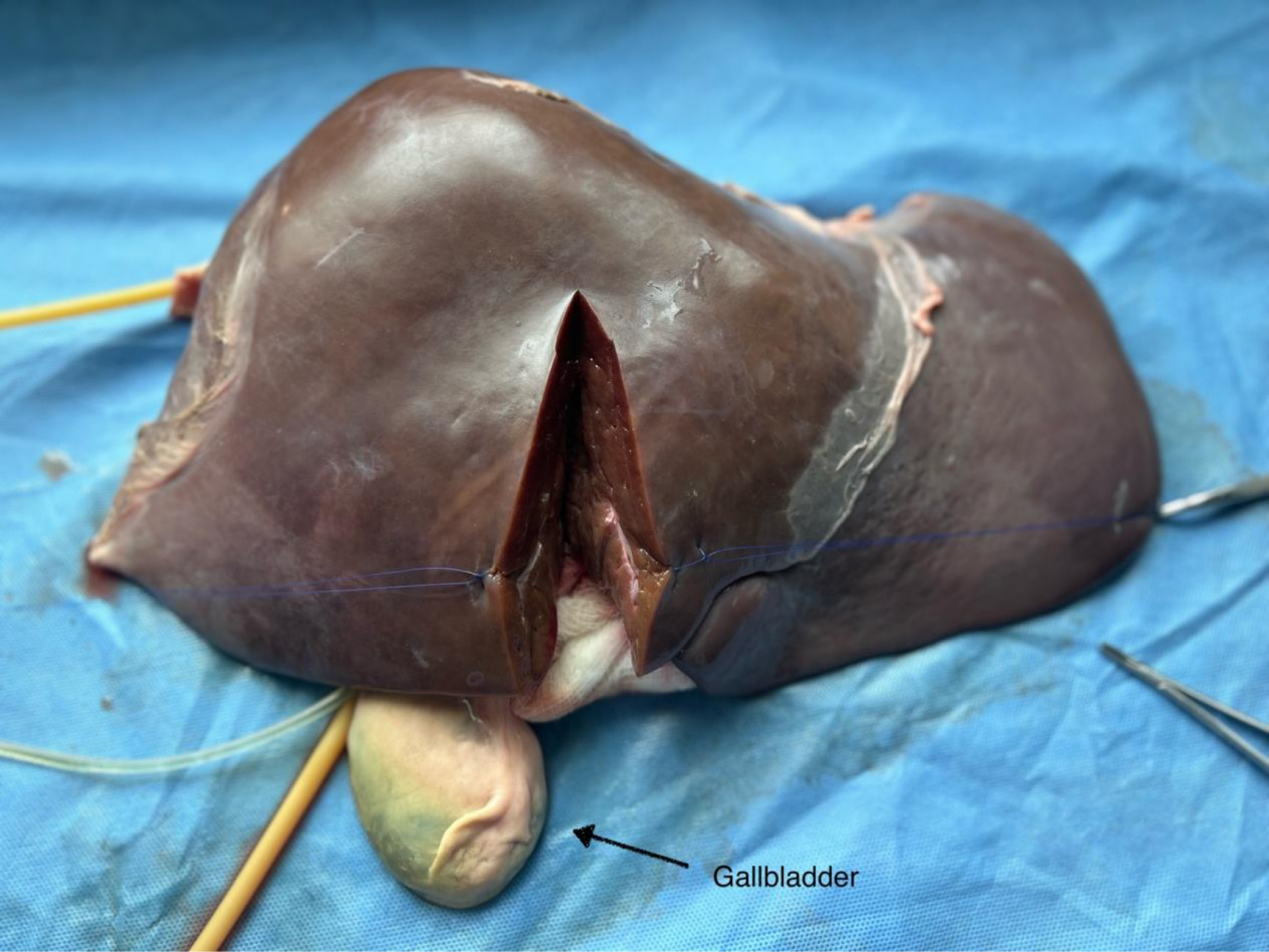
Right hepatectomy.

**Figure 4.**
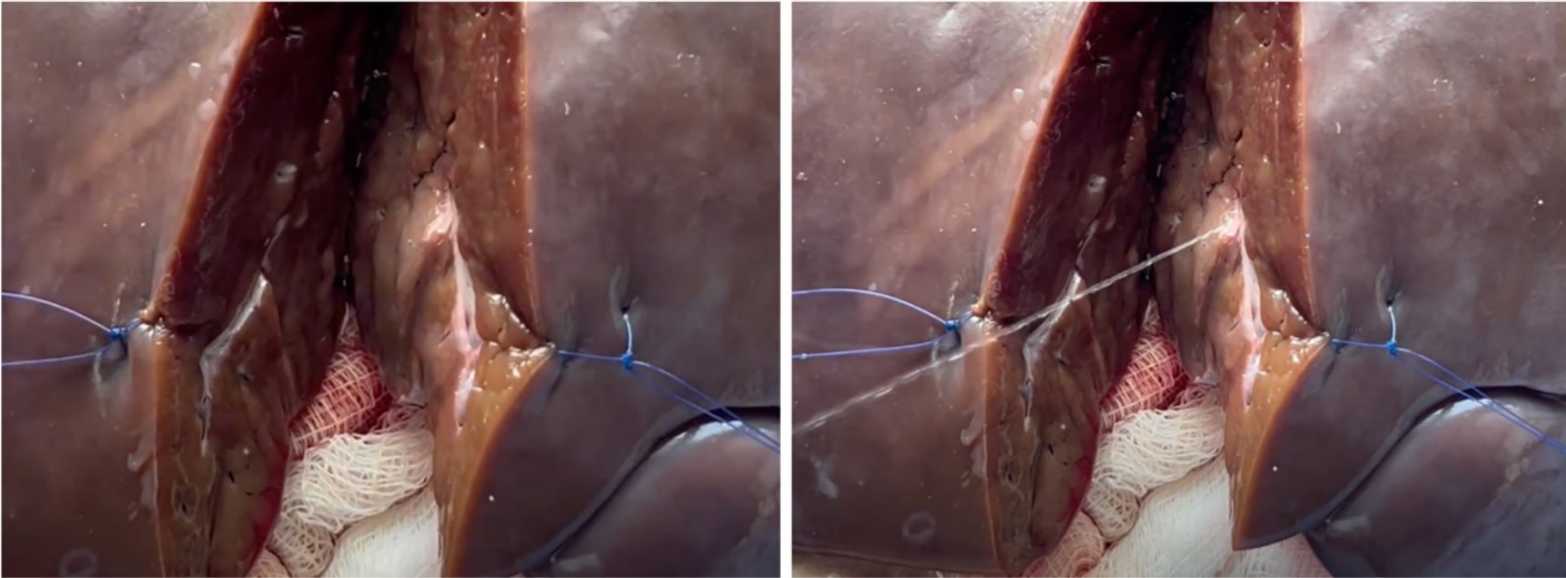
Arterial bleeding simulated by the laboratory manager intermittently injecting water through the 60 mL syringe.

The bovine liver was placed on a slanted tray that allowed the collection of fluid to leave the model. Both inflow and outflow volumes were recorded during the simulation to quantify an estimate of ‘blood loss’. The procedure performed in this simulation training was a right hepatectomy (Figure 3). The surgical instruments used were a 10-blade scalpel, suture scissors, Metzenbaum scissors and various clamps including a mosquito, Kelly clamp, right angle, needle drivers and forceps. The suture types included silk and prolene ties. Surgical skill exercises performed included an ultrasound of liver anatomy, planning the surgical resection plane, crush clamp parenchymal dissection, vessel suture ligation and suture stick-tying. Arterial bleeding was simulated by intermittently injecting water through the 60 mL syringe as exemplified in Figure 4, Supp. Video 1. Operative time, estimated blood loss, surgical technique, proficiency and critical decision-making in the face of inadvertent complications were assessed during the exercise.

## Results

**Figure 5.**
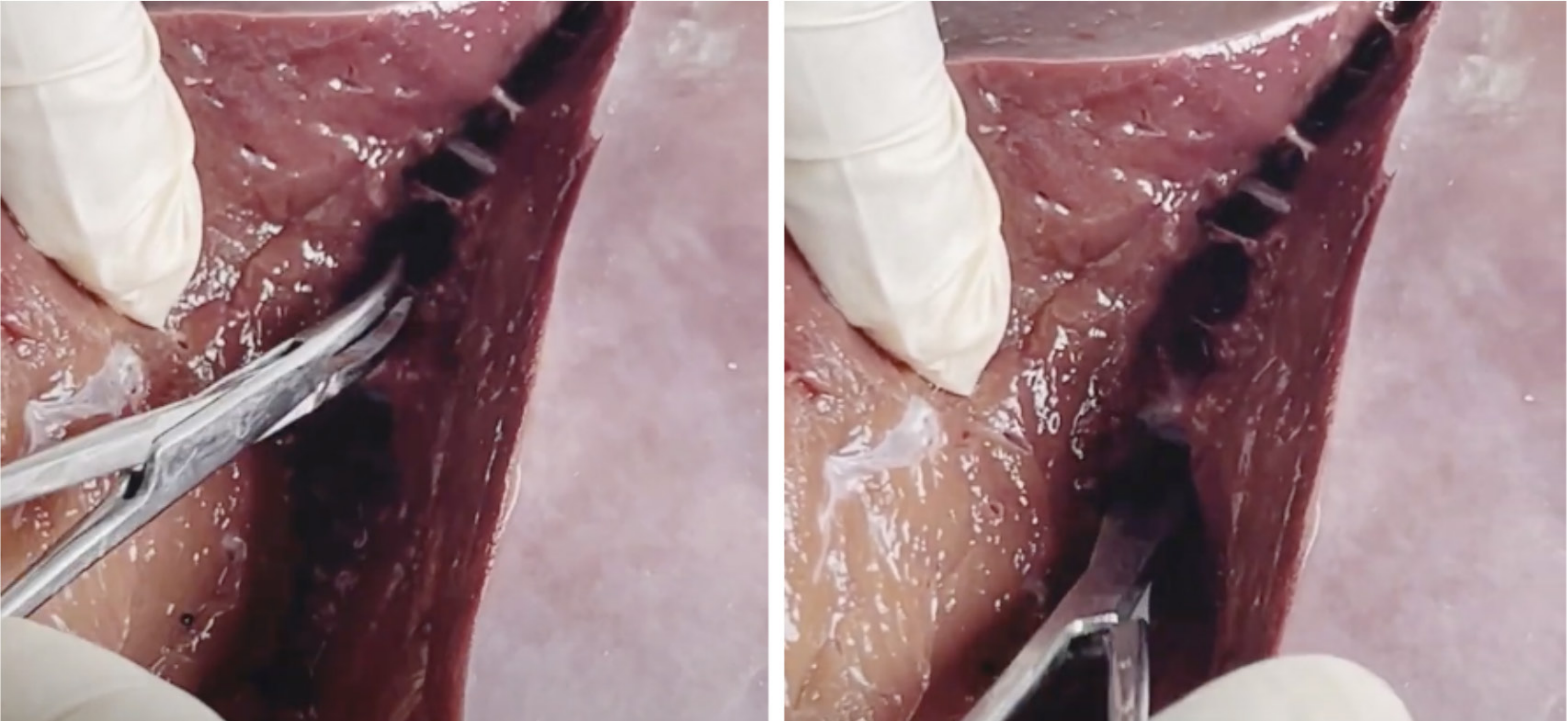
Parenchymal division, vessel handling, anatomic resection planning and simulated blood loss.

**Figure 6.**
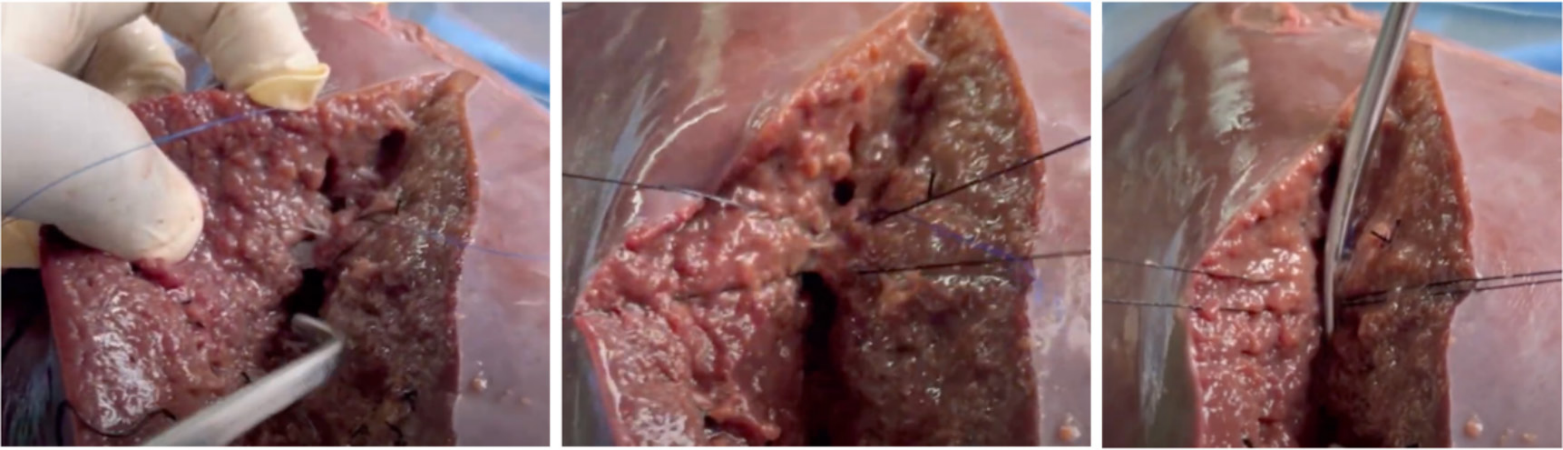
Simulated bleeding effectively managed using suture ligation techniques.

Creation of the bovine liver model cost a total of USD 82.66. Laboratory preparation of the model was completed in less than 1 hour. Training on model preparation was completed by surgical residents and the laboratory manager. Two experienced hepatobiliary surgeons performed a right hepatectomy on the bovine liver model. The model performed well in simulating parenchymal division, vessel handling, anatomic resection planning and simulated blood loss (Figure 5, Supp. Video 2). Hemodynamic alterations were successfully induced through controlled clamping and altering fluid container height to simulate portal hypertension or changes in CVP. Effects of the pringle maneuver were successfully replicated by simultaneously clamping the PV and PHA. Simulated bleeding was effectively managed using suture ligation and stick-tying techniques (Figure 6, Supp. Video 3).

## Discussion

HCC is the second leading cause of cancer-related mortality in SSA. Surgical hepatectomy plays a major role in early-stage HCC, as well as in other diseases such as colorectal liver metastasis, cholangiocarcinoma and liver cysts. The World Health Organisation estimates that by 2030, 60% of all new cancer cases will occur in LMICs, and although surgery is responsible for approximately 65% of all cancer cure and control, more than 75% of patients with cancer worldwide do not get safe, affordable or timely surgery. Therefore, innovative surgical training programs are needed to address the growing gap between disease and care providers [1].

Hepatobiliary surgical training is limited in SSA and other LMICs, partly due to a limited number of training facilities and experts despite the increasing demand. As a result, there is less exposure for faculty, and subsequently fewer training opportunities for fellows, surgical residents and medical students. This leads to suboptimal care for patients requiring such services. Therefore, we devised a quality, cost-effective, high-fidelity training model for liver surgery to be readily available for surgical skills training. We hypothesise that by creating an inexpensive and sustainable dry animal model for liver surgery in Tanzania, we can greatly impact surgical training in LMICs worldwide.

Simulation training in surgery can be divided into two main groups: inorganic and organic. Inorganic models include three-dimensional models made of silicone or plastic and virtual reality programs with haptic feedback, while organic models can be either human or animal. In recent years, high-income countries have devised three-dimensional models and virtual reality programs for surgical simulation [6, 7]. These models require resources and capital that may not be available in most parts of the world [8].

Regarding organic models, human models are generally supplied by cadavers donated to science and preserved in formalin. Anatomic accuracy of the human cadaver model leads to greater applicability in future clinical settings; however, the tissue quality is impaired by formalin preservation. Additionally, the reproduction of hemodynamic simulated alterations in real-time is limited. Animal models are, therefore, an alternative to human models and can be either ‘wet’ or ‘dry’. Wet animal models are live animals that have been anesthetised and subsequently euthanised once the training session is complete. This practice is largely viewed as unethical and is illegal in some countries. On the other hand, dry animal models refer to the use of animal parts extracted from slaughterhouses. This has been successfully applied in various surgical training programs in an LMIC, allowing surgeons to practice surgical techniques using porcine models [2, 9].

Efforts to develop effective animal models for liver surgery training have been previously described. In 2007, the use of sheep liver for laparoscopic liver resections was noted to have the advantage of similar anatomy to the human liver [10]. Similarly, in 2016, a Chinese research team created an *ex vivo* lamb liver model with continuous perfusion to simulate bleeding, offering a more realistic environment for laparoscopic simulation [11]. Trainees using this model performed significantly better than those using traditional dry box models, underscoring the value of animal models in providing realistic experiences that enhance surgical skills. Differing from these prior models, we found that the bovine liver best served our purposes in terms of availability, size and anatomy, as analogous anatomy is key to any surgical model. A 2003 study taking 20 bovine liver resin cast specimens illustrated the bovine liver as divided primarily using the intrahepatic portal system and secondarily by the hepatic veins, similar to how the human hepatic segments were determined [12].

This bovine liver model was trialled by senior faculty who performed liver surgery at Muhimbili National Hospital and it was unanimously determined to be a realistic experience in parenchymal dissection and anatomy. Our results demonstrate the feasibility of using a dry animal model for hepatobiliary surgery to increase exposure to surgical techniques in a realistic, inexpensive, sustainable and ethical manner in resource-constrained settings. We demonstrated that this model can be replicated to suit several training programs that will benefit surgical attendings, residents and students. Training exercises can range from practicing commonly used liver surgery techniques (such as crush-clamping and suture ligation) to simulating the pringle maneuver as described in this paper. Instructors can also simulate common situations encountered in actual surgical cases such as arterial bleeding and change in CVP. This will allow the instructors to evaluate participants’ responses to case-by-case variation and commonly encountered complications. Another assessment could be operative pace by timing how long it would take to complete the procedure on the model. We are developing a curriculum that will combine didactic lectures with this bovine liver surgery lab. We expect to pilot the curriculum with residents, fellows and junior surgeons at Muhimbili National Hospital. We will have pre- and post-assessments and questionnaires to test their knowledge of liver surgery and the surgical management of liver cancer overall.

Key considerations in future implementations of this training model may include ensuring an adequate water supply for the laboratory, as well as having surgical instruments and sutures readily available for procedures. During liver procurement from the stockyard, it is crucial to preserve the entire liver along with the major vessels. This will save time in preparation. The immediate flushing of the vessels is also essential to prevent coagulation of blood within the intrahepatic vessels, which could disrupt the water flow system and introduce potential errors. Finally, this is an intrahepatic dissection model and one of the limitations is that mobilising the liver and dissecting the retrohepatic IVC is not simulated in this model, in part due to different orientation of the IVC to the liver in bovine as compared to humans.

## Conclusion

This study demonstrates the successful design of a cost-effective and sustainable liver model for surgical training. Its practical design, low maintenance requirements and affordability—at approximately $80 per model—make it a valuable resource for hospitals and academic institutions in LMICs such as Tanzania. The model’s anatomy closely mirrors the human liver, making it suitable for teaching transection techniques, anatomical orientation and simulating different hemodynamic scenarios. Easily sourced and replicated using basic supplies commonly available in LMICs, this model provides an accessible and realistic platform for surgical skill development. Being adaptable to various settings, it holds the potential to improve surgical outcomes and enhance patient care in resource-limited environments.

## Conflicts of interest

The authors declare that they have no competing interests.

## Funding

None.

## Informed consent

Not applicable.

## Author contributions

Conceptualisation: AHM, CEG, JJ, SW

Methodology: AHM, CEG, JJ, SW

Writing – original draft: JJ

Writing – review and editing: JJ, CEG, DWK, AHM, NEK

All authors have approved the submitted manuscript.

## Trial registration

N/a.

## Figures and Tables

**Table 1.** Tools and cost used in developing the cow’s liver model.

Item	Units	Unit cost (USD)	Total cost (USD)
Cow liver	1	20·30	20·30
Plastic water bucket with spigot	2	16·61	33·22
½ inch Penrose drain	1 box	9·23	9·23
Food coloring/colored juice	2	5·54	11·08
Paper towels	2	2·95	5·90
Gloves	1 box	1·48	1·48
Intravenous line plastic tubing	1	0·55	0·55
Foley catheter	2	0·32	0·64
60 mL syringe	1	0·26	0·26
Surgical instruments	N/A	N/A	N/A
Sutures	N/A	N/A	N/A
Water	N/A	N/A	N/A
Total cost	USD 82·66		

N/A: Not applicable (Available in the Lab)

USD: United States Dollars

ml: milliliters

**Supplemental Video 1.**
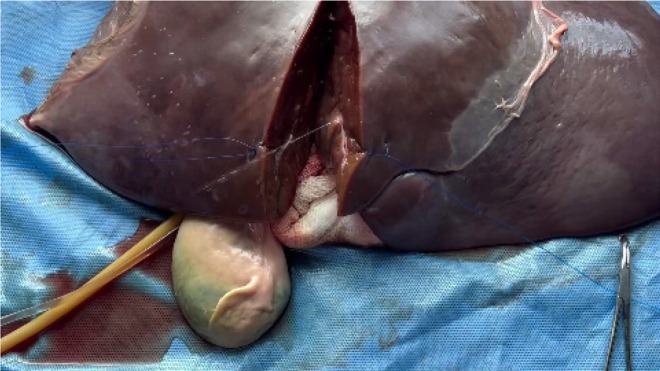
Arterial bleeding simulated by the laboratory manager intermittently injecting water through the 60 mL syringe. To view the video, visit https://vimeo.com/1087041097/8476f91b86?share=copy.

**Supplemental Video 2.**
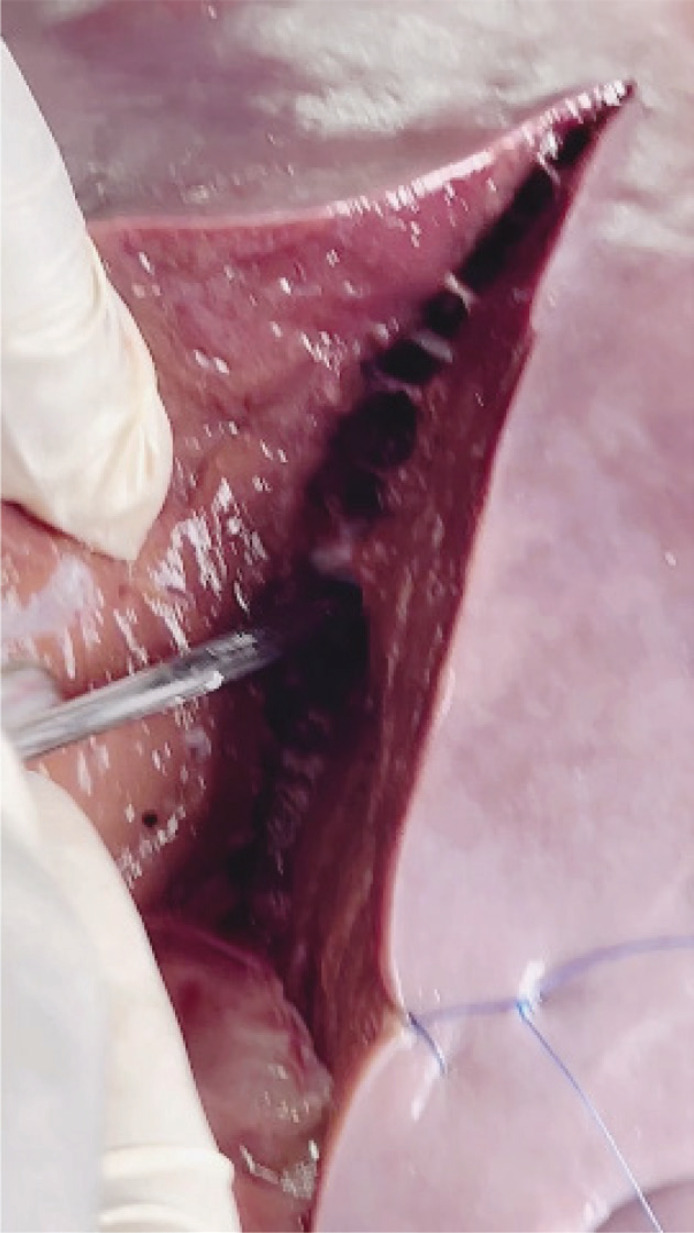
Parenchymal division, vessel handling, anatomic resection planning and simulated blood loss. To view the video, visit https://vimeo.com/1087041113/a95eb4927f?share=copy.

**Supplemental Video 3.**
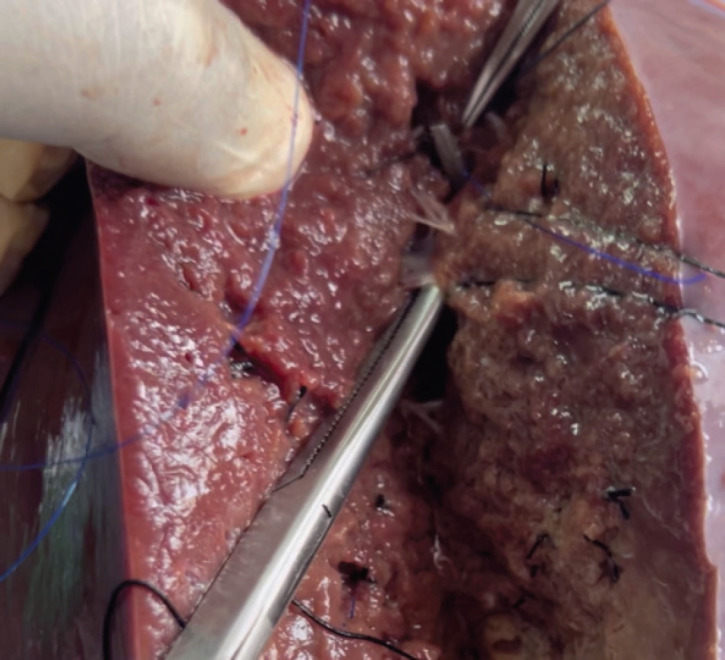
Simulated bleeding effectively managed using suture ligation techniques. To view the video, visit https://vimeo.com/1087041178/cd9cd2f7e0?share=copy.
